# High Red Cell Distribution Width and Low Absolute Lymphocyte Count Associate With Subsequent Mortality in HCV Infection

**DOI:** 10.20411/pai.v6i2.467

**Published:** 2021-10-07

**Authors:** Sofi Damjanovska, Perica Davitkov, Surya Gopal, Lenche Kostadinova, Corrine Kowal, Alyssa Lange, Anita Moreland, Carey L. Shive, Brigid Wilson, Taissa Bej, Sadeer Al-Kindi, Yngve Falck-Ytter, David A. Zidar, Donald D. Anthony

**Affiliations:** 1 Department of Medicine, Cleveland VA Medical Center, Case Western Reserve University; 2 Division of Gastroenterology, Cleveland VA Medical Center, Case Western Reserve University; 3 Department of Pathology, Case Western Reserve University, Cleveland, OH; 4 Research and Education Foundation for Cleveland VA, Cleveland, OH; 5 Department of Medicine, MetroHealth Medical Center, Cleveland, OH; 6 Department of Medicine, University Hospitals Cleveland Medical Center; 7 University Hospitals Harrington Heart and Vascular Institute, University Hospitals Cleveland Medical Center

**Keywords:** Red cell, Lymphocyte, Rheumatoid arthritis, Tumor Necrosis Factor, inflammation

## Abstract

**Background:**

Hepatitis-C virus (HCV) chronic infection can lead to cirrhosis, hepatocellular carcinoma (HCC), end-stage liver disease, cardiovascular disease (CVD), and mortality. Transient Elastography (TE) is used to non-invasively assess fibrosis. Whether immune monitoring provides additive prognostic value is not established. Increased red-cell distribution width (RDW) and decreased absolute lymphocyte count (ALC) predict mortality in those without liver disease. Whether these relationships remain during HCV infection is unknown.

**Materials and Methods:**

A retrospective cohort of 1,715 single-site VA Liver Clinic patients receiving Transient Elastography (TE) 2014-2019 to evaluate HCV-associated liver damage were evaluated for RDW and ALC in relation to traditional parameters of cardiovascular risk, liver health, development of HCC, and mortality.

**Results:**

The cohort was 97% male, 55% African American, 26% with diabetes mellitus, 67% with hypertension, and 66% with tobacco use. After TE, 3% were subsequently diagnosed with HCC, and 12% (n=208) died. Most deaths (n=189) were due to non-liver causes. The TE score associated with prevalent CVD, positively correlated with atherosclerotic cardiovascular disease (ASCVD) 10-Year Risk Score, age, RDW, and negatively correlated with ALC. Patients with anisocytosis (RDW above 14%) or lymphopenia (ALC level under 1.2×10^9^/L) had greater subsequent all-cause mortality, even after adjusting for age, TE score, and comorbidities. TE score, and to a modest degree RDW, were associated with subsequent liver-associated mortality, while TE score, RDW, and ALC were each independently associated with non-liver cause of death.

**Conclusion:**

Widely available mortality calculators generally require multiple pieces of clinical information. RDW and ALC, parameters collected on a single laboratory test that is commonly performed, prior to HCV therapy may be pragmatic markers of long-term risk of mortality.

## INTRODUCTION

The overall burden of liver cancer worldwide is increasing. Hepatocellular carcinoma (HCC) accounts for >80% of primary liver cancers [1], and it is estimated to be the fourth most common cause of cancer-related death worldwide [2]. The incidence of HCC in the United States has increased 2-3 fold over the past 3 decades [3]. Chronic hepatitis B (HBV) and hepatitis C virus (HCV) infection are the most common underlying causes of HCC [4].

Chronic HCV infection is a systemic disease, and its clinical impact and prognosis depend not only on morbidity and mortality related to liver disease, but also on extrahepatic manifestations. Chronic HCV infection results in a state of immune activation and inflammation evidenced by increased circulating interleukin 6 (IL-6), tumor necrosis factor-alpha (TNF-alpha), C-reactive protein (CRP), and fibrinogen [5].

Markers related to red-cell homeostasis, such as the red-cell distribution width (RDW), have previously been linked to liver disease, including outcomes related to HBV infection [6] [7], primary biliary cirrhosis [8], and non-alcoholic fatty liver disease [9]. An elevated RDW also predicts all-cause mortality, including cardiovascular disease (CVD) death [10] [11]. In colon cancer, a pre-operative RDW cut-off of 14 was identified using receiver operator characteristic curve analysis with an area under the curve of 0.704 [12]. An RDW above 14 was associated with a 4.2 odds hazard ratio for mortality over 4.5 years, independent of hemoglobin level and tumor-related features [12]. Anisocytosis can accompany anemia. Systemic inflammation can induce erythropoietin resistance and associated anemia. However, the mechanisms linking RDW to disease outcomes have not been established.

The absolute lymphocyte count (ALC) can also serve as a crude marker of immunologic homeostasis. Cirrhosis has been shown to be associated with lymphopenia, in particular abnormally low CD4 T-cell counts [13]. Our group has recently shown lymphopenia in the general population is associated with reduced longevity independently of traditional risk factors and RDW [10]. In a cohort with myelodysplastic syndrome, an ALC under 1.2 x 10^9^/L was associated with lower overall survival [11]. How RDW and ALC relate to inflammatory parameters, CVD risk, and mortality in chronic HCV has not been previously investigated.

Transient elastography (TE) is an ultrasound-based method used to identify patients with liver fibrosis [14]. Using a cut-off value of 12.5 kPa and above detects cirrhosis with 91% specificity and 87% sensitivity compared to liver biopsy [15]. Risk for developing HCC is greater in those with advanced fibrosis, and TE has allowed for stratification of risk for HCC. Notably, TE score reduces after direct-acting antiviral (DAA) therapy [16] [17] [18] [19], and it is thought that liver stiffness during chronic infection is a combination of hepatic inflammation and fibrosis. After viral eradication the component of inflammation in large part abates, followed by a slower phase of fibrosis regression.

Understanding relationships among TE score, ALC, RDW, HCC, and mortality may help guide the care of patients with chronic HCV infection, especially after successful HCV therapy. We performed a retrospective cohort study to evaluate relationships between RDW, ALC, liver damage, and mortality in the setting of chronic HCV infection.

## MATERIALS AND METHODS

### Study Participants and Data Extraction

In a retrospective cohort of 1,715 Cleveland VA Liver Clinic patients with chronic HCV infection who completed Transient Elastography (TE) between 2014 and 2019, we evaluated RDW and ALC at the time of the liver stiffness assessment under a VA IRB approved protocol. We examined the relationship between traditional parameters of liver function, subsequent HCC diagnosis, and subsequent mortality.

Charts were reviewed for demographic information, lipid profiles (high and low density lipoproteins and total cholesterol, with normal reference range 35-80 mg/dL; 0-110 mg/dL; 135-200 mg/dL; respectively), smoking history, ICD9/10 diagnosis of HCC, ICD9/10 diagnosis of hypertension (HTN), systolic and diastolic blood pressure, ICD9/10 diagnosis of diabetes mellitus (DM), hemoglobin (with reference range for males 13.6-17.4 g/dL), RDW (reference range 11.2-15.8%), ALC (reference range 0.8-5.0 10^9^/L), albumin (reference range 3.4-5.5 g/dL), platelet count (reference range 150-400 10^9^/L), and glucose (reference range 70-120 mg/dL) level. APRI (AST to Platelet Ratio Index) and FIB-4 (Fibrosis-4) score were calculated [20] [21]. Charts were reviewed for AST (Aspartate Aminotransferase, reference range <40 U/L), ALT (Alanine Aminotransferase, reference range <45 U/L), and AFP (Alpha Fetoprotein, reference range 8 ng/mL). Race was identified as black, Hispanic, white, or other.

Patient mortality and cause for mortality was collected. HCC diagnosis was confirmed by looking up the interdisciplinary tumor board note in the medical charts. Because these patients were regularly followed in liver clinic (every 3-6 months in most cases), unknown cause of death was considered unlikely to be liver related when there was no laboratory or clinical evidence of de-compensated cirrhosis (Child-Pugh Class B) or liver clinic progress notes indicating decompen-sated cirrhosis. Thus, we considered 2 causes of death categories in our analysis: liver vs non-liver, while other known causes of death are also discussed.

### Transient Elastography

Liver stiffness measurement was performed using transient elastography (FibroScan model 502, Echosens, France) as part of standard clinical care for evaluation of liver damage and fibrosis, most commonly for assessment prior to starting HCV therapy. Ten consecutive and successful measurements were performed on each patient and only those obtained with a success rate of at least 60% and an interquartile range/median value (IQR/M) less than 30% were considered reliable. The results were expressed in kilopascals (kPa). Cut-off of 12.5 kPa was used to determine cirrhosis.

### Statistics

Associations between continuous variables were assessed using Spearman rank sum correlations. Survival analyses, including log-rank tests and Cox proportional hazard models, were used to assess mortality across biomarker levels while adjusting for demographics, TE score, and cardiovascular risk. Cut-off points for RDW and ALC in survival analyses (1.2 10^9^/L for ALC and 14% for RDW) were based upon prior cohort survival analyses [12] [11]. Comparisons of continuous variables and categorical variables across independent groups were performed using the Mann-Whitney U and chi-square tests, respectively. All *P*-values presented are 2-sided and unadjusted. Statistical analyses were performed using SPSS for Windows v. 24.0 (IBM Corp, Armonk, New York) and R Statistical Software v 3.5.1 (R Core Team, Vienna, Austria).

### Results

#### Demographics and Clinical Parameters at the Time of Transient Elastography (TE)

Clinical features at the time of TE of the 1,715 patients with HCV infection that underwent TE at our single VA center are shown in Table 1. As expected for a VA cohort, the majority were male (97%), had hypertension (67%), and were active smokers (66%). Many had diabetes (26%) and were on statin therapy (38%). From the time of TE performance, median [IQR] of chart follow-up was 4 [3, 5] years for the whole group.

**Table 1.** Study Cohort Characteristics^*^

	All Patients	RDW ≤ 14	RDW > 14	ALC ≥ 0.2	ALC < 1.2
**Number**	1715	1163	525	1583	104
**Age (years)**	66	64	65	64	67
**Median (IQR)**	(60, 68)	(60, 68)	(61, 69)	(60, 68)	(63, 70)
**Expired patients n (%)**	208 (12%)	90 (8%)	61 (12%)	134 (9%)	17(16%)
**Race *Black***	55%	50%	68%	55%	58%
**Race *White***	41%	46%	31%	41%	40%
**Race *Hispanic***	3%	1%	0.5%	1%	2%
**Race *Other***	1%	3%	0.5%	3%	0%
**Sex *Male***	97%	97%	97%	97%	100%
**Sex *Female***	3%	3%	3%	3%	0%
**DM n (%)**	442 (26%)	283 (24%)	157 (30%)	405 (26%)	33 (32%)
**HTN M (%)**	1142 (67%)	756 (65%)	376 (72%)	1055 (67%)	73 (70%)
**cholesterol (mg/dL)**	156 (133, 180)	159 (132, 180)	153 (131, 179)	157 (133, 180)	137 (121, 162)
**LDL (mg/dL)**	90 (71, 111)	94 (73, 112)	88 (68, 110)	91 (71, 111)	91 (64, 94)
**HDL (mg/dL)**	46 (38, 57)	45 (37, 57)	46 (37, 59)	46 (38, 57)	42 (35, 52)
**Smoking**	current 66%; former 21%; no 13%	current 65%; former 22%; no 13%	current 68%; former 21%; no 11%	current 67%; former 21%; no 12%	current 56%; former 30%; no 14%
**ASCVD 10-Year Score (%)**	20% (13, 30)	20% (13, 30)	22% (15, 32)	20% (13, 29)	24% (16, 35)
TE Score (kPa)					
≤2.4	7	2	0	5	2
2.5–7.4	856	581	257	801	41
7.5–9.4	310	208	91	285	20
9.5–12.4	213	157	48	190	9
≥12.5	329	204	117	304	22
**Viral load (logl0)**	6.3 (5.7, 6.7)	6.2 (5.6, 6.7)	6.2 (5.6, 6.7)	6.2 (5.6, 6.7)	6.2 (5.8, 6.6)
**AFP (ng/mL)**	4.7 (3, 8.1)	4.5 (2.8, 7.8)	4.6 (3, 8.7)	4.7 (3, 8.1)	4.4 (2.6, 8.8)
**AST (U/L)**	38 (26, 56)	38 (28, 57)	35 (24, 52)	37 (26, 55)	44 (28, 68)
**ALT (U/L)**	48 (33, 75)	50 (35, 76)	43 (29, 66)	48 (33, 75)	48 (31, 75)
**HCC n (%)**	51 (3%)	32 (3%)	19 (4%)	49 (3%)	2 (2%)
**RDW (%)**	13.5(13, 14.2)	13.6(13.4, 13.8)	14.7(14.3, 15.4)	13.5(12.9, 14.2)	13.9(13.1, 14.7)
**ALC (×l0^9^/L)**	2.2 (1.7, 2.8)	2.2 (1.6, 2.8)	2.2 (1.6, 3)	2.2 (1.8, 2.9)	1.1 (0.9, 1.2)
**Albumin (g/dl)**	3.7(3.5, 4)	37(3.5, 4)	37(3.4, 3.9)	3.8(3.5, 4)	3.6(3.4, 3.8)
**PLT (×10^9^/L)**	206 (170, 251)	203 (167, 244)	214 (174, 262)	210 (173, 253)	176 (138, 207)
**APR!**	0.4 (0.3, 0.6)	0.4 (0.3, 0.6)	0.4 (0.3, 0.6)	0.4 (0.3, 0.6)	0.6 (0.4, 1.1)
**FIB-4**	1.9 (1.3, 3.3)	1.9 (1.3, 3.4)	1.8 (1.3, 3.4)	1.8 (1.3, 3.1)	3.1 (1.7, 6.8)
**Follow-up (years)**	4 (3, 5)	4 (3, 5)	3 (3, 4)	4 (3, 5)	3 (2, 5)

* Median (Q1, Q3) values are shown for clinical characteristics unless otherwise stated.

Patients who had prevalent CAD had a higher RDW and lower ALC when compared to patients who did not [median 13.7 (13.1; 14.4) vs 13.5 (12.9; 14.2), *P*=0.001, and median 2.03 (1.54; 2.58) vs 2.2 (1.69; 2.83), *P*=0.001, Table 2]. Additionally, patients who had CAD had higher TE scores [7.9 (6.3; 10.4) vs 7.3 (5.3; 10.9)]. Other parameters that differed between those with vs without CAD included age, hemoglobin, PLT, glucose, albumin, and APRI (Table 2). Similarly, patients who had HTN had higher RDW when compared to patients who did not [median 13.6 (13; 14.3) vs 13.4 (12.9; 14.1), *P*=0.001, not shown].

**Table 2.** Parameters at time of TE associated with CAD^*^

Coronary Artery Disease (CAD)			
	no (n = 1422)	yes (n = 289)	p value
**Age (years) Median (IQR)**	64 (60, 67)	67 (63, 70)	<0.0001
**TE Score (kPa)**	7.3 (5.3, 10.9)	7.9 (6.3, 10.4)	0.002
**AST (U/L)**	37 (26, 55.25)	38.5 (27, 57)	0.578
**ALT (U/L)**	48 (33, 78)	49 (32.25, 74.75)	0.718
**ALC (×10^9^/L)**	2.2 (1.685, 2.83)	2.025 (1.54, 2.58)	0.001
**hbg (g/dl)**	14.6 (13.5, 15.525)	14.2 (13.1, 15.3)	0.002
**RDW (%)**	13.5 (12.9, 14.2)	13.7 (13.1, 14.4)	0.001
**PLT (×l0^2^mm^2^)**	210 (172.5, 254)	192 (160, 237)	<0.0001
**glucose (mg/dl)**	97 (87, 112)	98.5 (88, 125.75)	0.03
**Albumin (g/dl)**	3.8 (3.5, 4)	3.7 (0.308, 0.677)	<0.0001
**APRI**	0.412 (0.281, 0.628)	0.482 (0.308, 0.677)	0.011
**FIB-4**	1.821 (1.27, 3.367)	2.128 (1.402, 3.126)	.202

* Median (Q1, Q3) values are shown for clinical characteristics unless otherwise stated.

**Figure 1.**
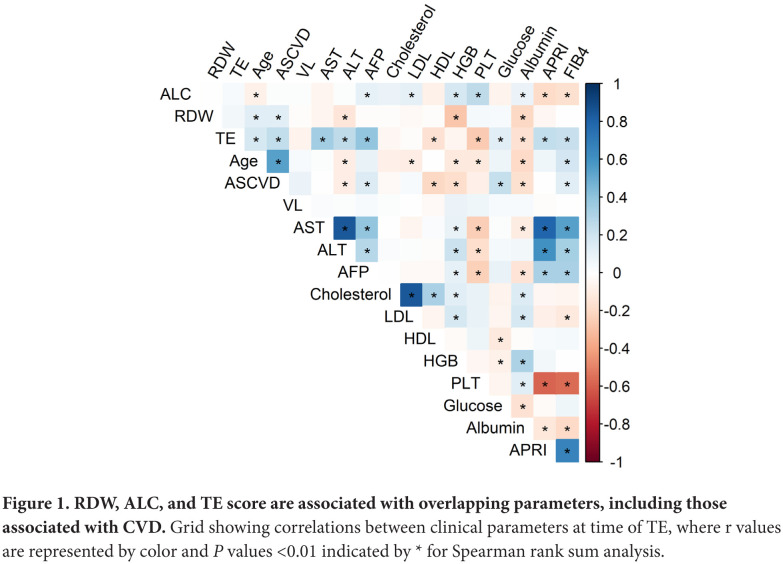
**RDW, ALC, and TE score are associated with overlapping parameters, including those associated with CVD.** Grid showing correlations between clinical parameters at time of TE, where r values are represented by color and *P* values <0.01 indicated by * for Spearman rank sum analysis.

Age was positively correlated with TE score (r=0.2, *P*<0.0001), RDW (r=0.1, *P*<0.0001), and negatively correlated with ALC (r = −0.1, *P*=0.009) and hemoglobin levels (r= −0.1, *P*<0.0001), though r values for these relationships were notably modest (Figure 1). Also, as expected, age was significantly higher in persons with a diagnosis of DM [65 (62; 69) vs 64 (60; 67) *P*<0.0001, Mann-Whitney U] or HTN [65 (61; 69) vs 62 (58: 66) *P*<0.0001, Mann-Whitney U], and negatively correlated with platelet count (r= −0.1, *P*<0.0001) and albumin (r= −0.2, *P*<0.0001).

Notably, TE, RDW, and ALC were correlated with an overlapping but different set of parameters. TE score correlated with age, ASCVD 10-Year Risk Score, AST, ALT, AFP, HDL, PLT, glucose, Alb, APRI, and FIB-4 (Figure 1). TE score also correlated with RDW (r=0.1, *P*=0.02, above *P* value threshold marked in Figure 1). At the same time RDW correlated with age, ASCVD score, ALT, Hgb, and Alb, while ALC correlated with age, AFP, LDL, Hgb, PLT, Alb, APRI, and FIB-4 (Figure 1).

#### TE score, RDW, and ALC are Each Independently Associated with Survival in Those with Hepatitis C

**Figure 2.**
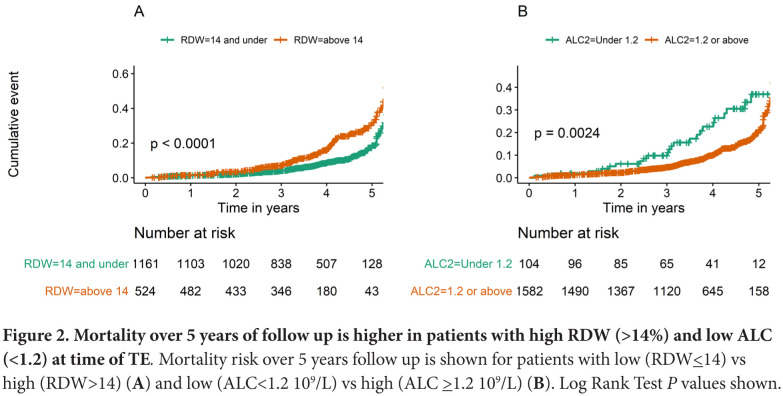
**Mortality over 5 years of follow up is higher in patients with high RDW (>14%) and low ALC (<1.2) at time of TE.** Mortality risk over 5 years follow up is shown for patients with low (RDW≤14) vs high (RDW>14) (**A**) and low (ALC<1.2 10^9^/L) vs high (ALC≥1.2 10^9^/L) (**B**). Log Rank Test *P* values shown.

At the time of performance of TE, patients who had anisocytosis (RDW above 14%) or lymph-openia (ALC level under 1.2×10^9^/L) had lower subsequent survival (log rank test *P*<0.0001 and *P*=0.002, respectively) (Figure 2). These survival differences persisted in a Cox proportional hazards model adjusting for age, sex, race, and ASCVD score (RDW HR=1.84, *P*<0.001; ALC HR=1.68, *P*=0.02) (Table 3), indicating that RDW and ALC are likely predictive of mortality in the setting of chronic HCV infection. Additionally, after adding TE score to the model, ALC and RDW were still significant predictors of mortality with HRs virtually unchanged (1.81 for RDW>14 and 1.66 for ALC<1.2, with *P*<0.001 and *P*=0.026, respectively) (Table 3). Furthermore, persons with high TE score had higher subsequent mortality, even after adjusting for age, sex, race, and ASCVD score (Table 3 and Supplemental Figure 1).

**Table 3.** Adjusted models for relationship between RDW, ALC, TE, and mortality

Model variables	Univariate models		Model 2 (adjusted)^*^		Model 3 (adjusted with TE)^**^	
	HR (95% Cl)	*P*-value	HR (95% Cl)	*P*-value	HR (95% Cl)	*P*-value
**RDW > 14**	1.94 (1.47, 2.56)	<0.001	1.84 (1.38, 2.45)	<0.001	1.81 (1.36, 2.42)	<0.001
**ALC < 1.2**	1.93 (1.25, 2.99)	0.003	1.68 (1.08, 2.61)	0.022	1.66 (1.06, 2.58)	0.026
**ASCVD; Elevated (7.5–20) vs. Low (<7.5) risk**	1.33 (0.57, 3.13)	0.512	0.98 (0.41, 2.37)	0.963	0.91 (0.37, 2.2)	0.828
**Age (years)**	1.03 (1.01, 1.05)	0.012	1.02 (0.99, 1.04)	0.183	1.01 (0.99, 1.04)	0.224
**Female**	0.56 (0.21, 1.52)	0.257	0.75 (0.27, 2.05)	0.571	0.74 (0.27, 2.03)	0.562
**Race Black vs. White**	1.01 (0.76, 1.33)	0.958	0.81 (0.61, 1.09)	0.161	0.84 (0.63, 1.12)	0.236
**TE score ≥ 12.5**	1.89 (1.37, 2.6)	<0.004			1.77 (1.27, 2.45)	0.001

* *Adjusted for race, sex, age, ASCVD*

** *Adjusted for race, sec, age, ASCVD and TE*

Of the 208 mortality events, 19 (9%) were attributable to liver-related causes of death (including HCC), while cancer (n=43, other than HCC), CVD (n=22), COPD (n=8), end-stage renal disease (n=5), sepsis (n=5), and non-liver unknown (n=106, chart information inconclusive but not consistent with liver disease causes accounted for the remainder). Compared to those with non-liver-related death, those persons who sustained liver-related mortality had higher baseline AST (*P*=0.01, Mann-Whitney U) and APRI (*P*=0.03, Mann-Whitney U). Considering all patients and using competing-hazards survival methods, we found baseline ALC<1.2 and RDW>14 to be associated with subsequent non-liver mortality (log rank test *P*=0.01 and *P*<0.001, respectively), RDW>14 was marginally associated with subsequent liver mortality (log rank test *P*=0.04), but RDW as a continuous variable was not associated with liver mortality after adjusting for age (Supplemental Figure 2). TE score >12.5 kPa was associated with both liver and non-liver mortality (*P*=0.003 and *P*=0.006).

Certainly, several liver-related mortality events were attributable to HCC. When focusing on HCC, after TE scan performance 51 patients were subsequently diagnosed with HCC, median 1 (0.4, 2.2) year after TE score was obtained. Age, DM, HTN diagnosis, statin use, TE score, AFP, ASCVD 10-Year Risk Score, platelet count, albumin, APRI, and FIB-4 all differed at the TE procedure time point comparing those subsequently diagnosed with HCC to those not diagnosed with HCC (Supplemental Table 1). RDW and ALC did not differ between HCC and non-HCC subgroups at time of TE.

## DISCUSSION

Here in this single-site cohort of the VA Liver Clinic, 1,715 patients received Transient Elastography (TE) during the time period 2014-2019 for evaluation of HCV-associated liver damage, and we observed RDW and ALC at time of TE to associate with subsequent all-cause mortality, a finding that remained after adjusting for age, TE score, and comorbid conditions. Additionally, TE score itself was associated with all-cause mortality. While TE score was found to associate with liver and non-liver causes of death, ALC and RDW appeared to be mainly associated with non-liver cause of death. We further found TE score to associate with CAD diagnosis, and correlate with ASCVD 10-Year Risk Score, age, and RDW, though the strength of the latter correlation was modest. Overall, these findings suggest that immunohematologic dysfunction prior to HCV therapy predicts subsequent all-cause mortality, likely through pathways associated with non-liver-related mortality. Widely available mortality calculators generally require multiple pieces of clinical information, including lab work data ideally collected at the same time. RDW and ALC prior to HCV therapy may be pragmatic markers of long-term risk of mortality. These 2 parameters collected with 1 lab test are cheap and frequently performed during standard clinical care.

In this study, the ALC and RDW are each age related, and thus lymphopenia and anisocytosis may be markers of accelerated immunologic aging. However, we find that RDW and ALC do not correlate with each other and the associated risk for all-cause mortality of each marker is independent of each other. Anisocytosis is associated with clinically derived ASCVD risk and is related to anemia and hypo-albuminemia [10] [22].

Unlike RDW, the ALC did not correlate with ASCVD risk score but was related to platelet counts, APRI, and FIB4. Taken together, we conclude that a high RDW and low ALC may identify pathways and mechanisms of adverse immunohematologic aging which are in part separable, and each distinct from TE score.

RDW reflects the variability in circulating red blood cell (RBC) size. It is based on the width of the RBC volume distribution curve, with larger values indicating greater variability. RDW is elevated when there is red cell loss, destruction, or, more commonly, ineffective red cell production. RDW may reflect nutritional deficiency (eg, iron, vitamin B12, or folic acid), bone marrow depression, and/or chronic inflammation. These conditions are often present in patients with liver disease, correlate with the severity of the disease, and are associated with a worse prognosis [23]. Higher RDW values were found in one study to be an independent predictor of mortality in HBV-infected patients [6], raising the possibility that RDW is associated with cirrhosis and/or portal hypertension. Here we observed at the time of performance of TE that persons with RDW of 14% or above had lower subsequent survival probability, even after adjusting for age, TE score, sex, race, and ASCVD score. We have previously shown that the RDW correlates with pro-inflammatory cytokines [24], though the specific mechanism for the RDW association with survival remains unclear [25–27]. Immunologic parameters associated with cirrhosis (sCD14, ATX, Mac2BP, MCP-1) might be expected to associate with RDW since RDW was found here to associate with TE score (a measure of fibrosis). While we attempted immunologic sampling in a small subset of HCC patients in the present study and did observe MCP-1 to correlate with RDW in persons with HCC (r=0.5, *P*=0.04, n=21), the sample size was too small to make firm conclusions. In this regard, future studies may lend insight into relationships that may be causal in this regard.

Prior studies have shown that lymphopenia, or a low peripheral blood lymphocyte count, may be a harbinger of greater mortality from advanced carcinomas, sarcomas, lymphomas, and even curable breast cancer [28] [29]. It has been shown that in septic patients with severe persistent lymphopenia there is significantly higher incidence of secondary infections and mortality [30]. In alcoholic hepatopathies there is impaired T-cell and antigen-presenting cell activation that leads to subdued response to bacterial and viral infections (increased susceptibility to tuberculosis, bacterial pneumonias, HIV, HCV) [31]. In liver transplant patients postoperatively the preoperative low ALC was associated with increased risk of postoperative infections [32]. Low ALC in the first postoperative month after liver resection due to HBV-related HCC was shown to be associated with a higher recurrence incidence [33]. Posttransplant ALC is associated with HCV recurrence after liver transplant as well [34]. Here, in our patients with lymphopenia and chronic HCV infection, we also observed increased all-cause mortality as well.

A prior meta-analysis found a 2-fold higher risk of subclinical carotid plaques among HCV-infected individuals compared with uninfected controls, as well as increased risk of carotid thickening [35]. Based on data from national surveys, sampled from the general population of Canada and the US, it is thought that the 10-year CVD risk is 2.5%-3.5% higher in HCV-infected persons [36]. In our single-site cohort, we found patients who had higher TE scores were more likely to have CAD, and their TE score correlated with their 10-Year ASCVD Risk Score and RDW.

Ten to twenty percent of patients with chronic HCV develop liver fibrosis or cirrhosis within 2 decades [37]. Chronic inflammation, immune-mediated hepatocellular destruction, and liver regeneration underlie cirrhosis, and are thought to play central roles in primary carcinogenesis. Once cirrhosis is diagnosed, HCC develops at a rate between 1.5% and 8% per year [38]. Here our subgroup with mortality (n=208) was composed of only a few with liver-associated cause of death (n=19), and only 51 (3%) were diagnosed over the follow-up time period with HCC. Therefore, our ability to evaluate factors associated with liver vs non-liver cause of death are limited by the small number of liver events. The low number of liver-related deaths is likely in part related to the non-random sampling of chronic HCV infected patients performed here, all having TE as the entry point to this cohort. The latter is a strength in making the population more uniform, nearly all were under evaluation for liver damage during chronic HCV infection. Since they were likely under evaluation for liver damage it is also likely there was no prior knowledge of cirrhosis at the time of TE, a scenario where decompensated liver function, HCC, or mortality are unlikely within the 5-year time window of follow up. Another limitation of this study was that it is retrospective in nature, and the majority of participants are male, as are most of the veteran population. Additionally, we did not consider sickle cell trait in our analysis, but we did evaluate whether associations with RDW were dependent on hemoglobin, and they were not. Finally, we are limited in ability to identify whether substance or alcohol contributes to altered RDW/ALC in this cohort. However, a criterion for HCV treatment in our liver clinic intake is no active substance or heavy alcohol use. We therefore believe “reported” heavy alcohol use would not be a factor. Despite these limitations, these data indicate TE score and RDW at baseline associate with subsequent liver cause of death, while ALC does not. At the same time, TE score, RDW, and ALC associate with non-liver cause of death, suggesting that ALC reflects non-liver pathways associated with mortality. Future study is needed to better understand linkages between RDW, ALC, and HCC to help guide post-HCV therapy management strategies.

## SUPPLEMENTARY DATA

Supplementary materials are available at the Pathogens and Immunity website. Supplementary data may be provided by the authors to benefit the reader. Supplementary data are not copyedited and are the sole responsibility of the authors. Questions or comments related to supplementary materials should be addressed to the corresponding author
