# Supplementary material for: High Red Cell Distribution Width and Low Absolute Lymphocyte Count Associate With Subsequent Mortality in HCV Infection
Source: Pathog Immun. 2021 Oct 7;6(2):90–104. doi: 10.20411/pai.v6i2.467 (PMC8714176; doi:10.20411/pai.v6i2.467)
Supplement: Supplemental Tables 1 [file pai-6-090-s01.pdf]

PATHOGENS AND IMMUNITY
IN ASSOCIATION WITH CASE WESTERN RESERVE UNIVERSITY AND THE RICHARD J. FASENMYER FOUNDATION

Supplemental Table 1. Parameters that differ by subsequent HCC status

	Hepatocellular carcinoma (HCC)		
	no	yes	p value
Age (years) Median (IQR)	64 (60, 68)	65 (63, 69)	0.017
TE Score (kPa)	7.4 (5.4, 10.4)	14 (9, 27.7)	<0.0001
AST (U/L)	37 (26, 55)	62 (44.5, 101.5)	<0.0001
ALT (U/L)	48 (33, 74)	78 (42, 110)	<0.0001
ALC (x10^9/L)	2.2 (1.66, 2.805)	2.1 (1.575, 2.96)	0.636
hgb (g/dl)	14.5 (13.4, 15.5)	14.3 (13.5, 15.5)	0.556
RDW (%)	13.5 (12.9, 14.2)	13.6 (13.2, 14.3)	0.267
PLT ($x10^3/mm^3$)	208 (171, 253)	162.5 (116.25, 202.25)	<0.0001
glucose (mg/dl)	97 (87, 113)	117 (88, 162)	0.019
Albumin (g/dl)	3.7 (3.5, 4)	3.5 (3.3, 3.8)	0.001
APRI	0.423 (0.282, 0.64)	0.59 (0.434, 0.754)	0.001
FIB-4	1.842 (1.281, 3.213)	3.612 (2.415, 5.755)	<0.0001

*Median (Q1, Q3) values are shown for clinical characteristics unless otherwise stated.

Damjanovska S, Davitkov P, Gopal S, Kostadinova L, Kowal C, Lange A, Moreland A, Shive CL, Wilson B, Bej T, Al-Kindi S, Falck-Ytter Y, Zidar DA, Anthony DD. High Red Cell Distribution Width and Low Absolute Lymphocyte Count Associate with Subsequent Mortality in HCV infection. *Pathogens and Immunity.* 2021;6(2): 90-104. doi: 10.20411/pai.v6i2.467
