# Supplementary material for: High Red Cell Distribution Width and Low Absolute Lymphocyte Count Associate With Subsequent Mortality in HCV Infection
Source: Pathog Immun. 2021 Oct 7;6(2):90–104. doi: 10.20411/pai.v6i2.467 (PMC8714176; doi:10.20411/pai.v6i2.467)
Supplement: Supplemental Figure 1 [file pai-6-090-s02.pdf]

PATHOGENS AND IMMUNITY
IN ASSOCIATION WITH CASE WESTERN RESERVE UNIVERSITY AND THE RICHARD J. FASENMYER FOUNDATION

Supplemental Fig 1

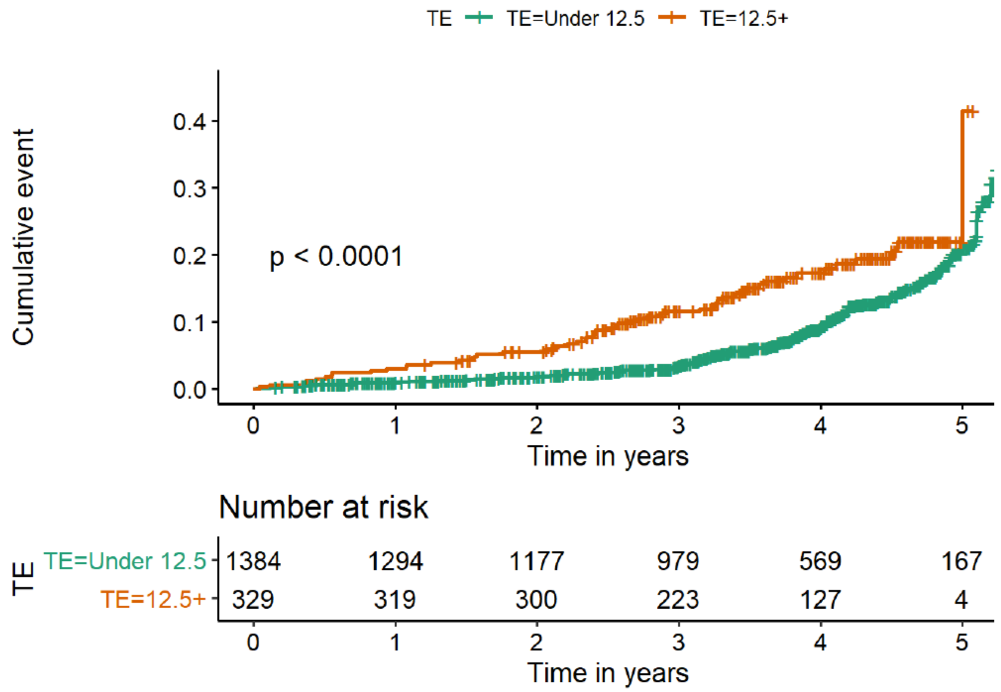

Supplemental Figure 1. High TE score is associated with all-cause mortality.

Mortality risk over 5 years of follow up is shown for patients with low (TE<12.5 kPa) vs. high (TE≥12.5 kPa) TE score.

Damjanovska S, Davitkov P, Gopal S, Kostadinova L, Kowal C, Lange A, Moreland A, Shive CL, Wilson B, Bej T, Al-Kindi S, Falck-Ytter Y, Zidar DA, Anthony DD. High Red Cell Distribution Width and Low Absolute Lymphocyte Count Associate with Subsequent Mortality in HCV infection. *Pathogens and Immunity.* 2021;6(2): 90-104. doi: 10.20411/pai.v6i2.467
