# Supplementary material for: High Red Cell Distribution Width and Low Absolute Lymphocyte Count Associate With Subsequent Mortality in HCV Infection
Source: Pathog Immun. 2021 Oct 7;6(2):90–104. doi: 10.20411/pai.v6i2.467 (PMC8714176; doi:10.20411/pai.v6i2.467)
Supplement: Supplemental Figure 2 [file pai-6-090-s03.pdf]

PATHOGENS AND IMMUNITY
In Association with Case Western Reserve University and The Richard J. Fasenmyer Foundation

Supplemental Fig 2

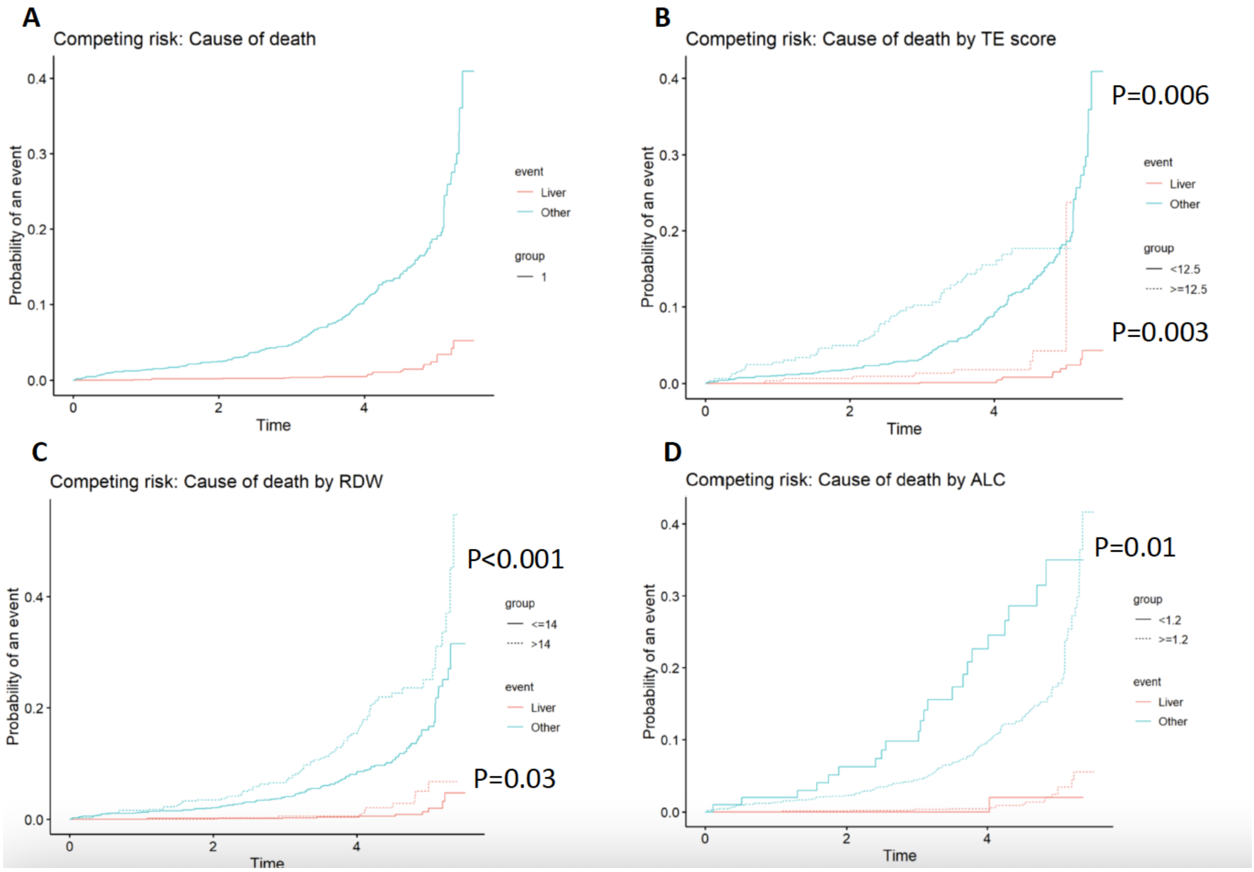

Supplemental Figure 2. High TE score and RDW are associated with both non-liver and liver cause of death, while low ALC is associated with non-liver cause of death. Mortality risk over 5 years of follow up is shown for patients with liver vs. non-liver cause of death (**A**), low (TE<12.5 kPa) vs. high (TE≥12.5 kPa) TE score by liver vs. non-liver cause of death (**B**), low (RDW≤14) vs. high (RDW>14) RDW by liver vs. non-liver cause of death (**C**), and low (ALC<1.2 K/cmm) vs. high (ALC≥1.2 K/cmm) ALC by liver vs. non-liver cause of death (**D**). Log Rank Test p values shown.

Damjanovska S, Davitkov P, Gopal S, Kostadinova L, Kowal C, Lange A, Moreland A, Shive CL, Wilson B, Bej T, Al-Kindi S, Falck-Ytter Y, Zidar DA, Anthony DD. High Red Cell Distribution Width and Low Absolute Lymphocyte Count Associate with Subsequent Mortality in HCV infection. *Pathogens and Immunity.* 2021;6(2): 90-104. doi: 10.20411/pai.v6i2.467
